# Underwater endoscopic mucosal resection for rectal neuroendocrine tumors (with videos): a single center retrospective study

**DOI:** 10.1186/s12876-022-02350-6

**Published:** 2022-06-02

**Authors:** Haitao Shi, Chuying Wang, Jie Wu, Bin Qin, Jiong Jiang, Na Liu, Yahua Song, Yun Qin, Shiyang Ma

**Affiliations:** 1 Department of Gastroenterology, The Second Affiliated Hospital of Xi’an Jiaotong University, 157 Xiwu Street, Xi’an, 710004 Shaanxi China; 2 Department of Pathology, The Second Affiliated Hospital of Xi’an Jiaotong University, Xi’an, 710004 Shaanxi China

**Keywords:** Endoscopic submucosal dissection (ESD), Rectal neuroendocrine tumor (rectal NETs), Underwater endoscopic mucosal resection (UEMR)

## Abstract

**Background:**

Underwater endoscopic mucosal resection (UMER) is a new method of endoscopic resection to completely remove the lesion without submucosal injection. But few attempts have been carried out for rectal neuroendocrine tumors (rectal NETs).

**Methods:**

We retrospectively investigated data on the tumor characteristics and outcomes of patients with ≤ 10 mm rectal NETs who underwent UEMR or endoscopic submucosal dissection (ESD) from January 2019 to June 2021 in our institute.

**Results:**

The endoscopic resection rate was 100% in both UEMR and ESD groups. The histological complete resection rate of the UEMR group (95.5%) was lower than that of the ESD group (96.4%) with no significant difference. The average operation time, hospitalization time and operation cost of UEMR group were less than those of ESD group (*P* < 0.05). The incidence of postoperative abdominal pain and abdominal distention in the UEMR group was lower than that in the ESD group (*P* < 0.05). There was no significant difference in the incidence of delayed bleeding and perforation between the two groups. There was no local recurrence or distant metastasis in the two groups during the follow-up period.

**Conclusions:**

Both the UEMR and ESD can effectively treat ≤ 10 mm rectal NETs with invasion depth confined to the mucosa and submucosa. UEMR is superior to ESD in operation time, hospitalization time, operation cost, postoperative abdominal pain and abdominal distention.

**Supplementary Information:**

The online version contains supplementary material available at 10.1186/s12876-022-02350-6.

## Background

Neuroendocrine neoplasms (NENs) are a group of heterogeneous tumors originating from peptidergic neurons and neuroendocrine cells, which can occur in many organs and tissues. According to the degree of differentiation, NENs are divided into well differentiated neuroendocrine tumors (NETs) and poorly differentiated neuroendocrine carcinoma (NEC). Among them, gastroenteropancreatic neuroendocrine neoplasms (GEP-NENs) are the most common, accounting for approximately 65% ~ 75% of all NENs [1]. In recent years, with the development of diagnostic endoscopy such as gastrointestinal endoscopy and endoscopic ultrasonography (EUS), the detection rate of GEP-NENs has gradually increased, and the rectum is the most common site for GEP-NENs [2]. Rectal neuroendocrine neoplasms (rectal NETs) are mostly non-functional with no clinical symptoms. Some non-specific symptoms such as pain, blood in the stool, and perianal discomfort were reported in patients with a relatively larger tumor [3]. Under the endoscopy, rectal NETs are mostly manifested as rectal polypoid masses. The probability of lymph node metastasis in ≤ 10 mm rectal NETs is low [4], which is around 1–4%. The clinical guideline suggests that endoscopic resection should be considered for NETs < 2 cm. For patients with an incomplete resection or a pathological G3 grade, surgery should be performed according to the norms of colon adenocarcinoma [5]. Endoscopic resection includes endoscopic mucosal resection (EMR) and endoscopic submucosal dissection (ESD) [5]. ESD prone to postoperative complications such as bleeding or perforation, also requires a high operation skill and a long operation time. In comparation, EMR may fail to remove submucosal tumor completely. Underwater endoscopic mucosal resection (UEMR) is a new method that subverts the traditional EMR operation. After the intestinal cavity is fulfilled with water, the UEMR can completely remove the lesion without submucosal injection. According to multi-center studies and meta-analysis, the en bloc resection rate of UEMR for colorectal lesions is significantly higher than that of the conventional EMR group, and does not increase the incidence of complications [6]. The purpose of this study is to explore the feasibility and outcome of UEMR in the treatment of rectal NETs and to provide reference for clinical treatment options.

## Methods

### Inclusion and exclusion criteria

Inclusion criteria: (1) Maximum tumor diameter ≤ 10 mm, examined with endoscopic ultrasonography before resection; (2) Diagnosed as neuroendocrine tumor by histopathological examination after resection; (3) Postoperative follow-up for more than 6 months. Exclusion criteria: (1) Metastasis; (2) Depth of tumor invasion exceeds submucosa revealed by endoscopic ultrasonography; (3) Severe cardiopulmonary disease, blood disease and blood coagulation dysfunction.

### Endoscopic procedures

#### UEMR

**Fig. 1:**
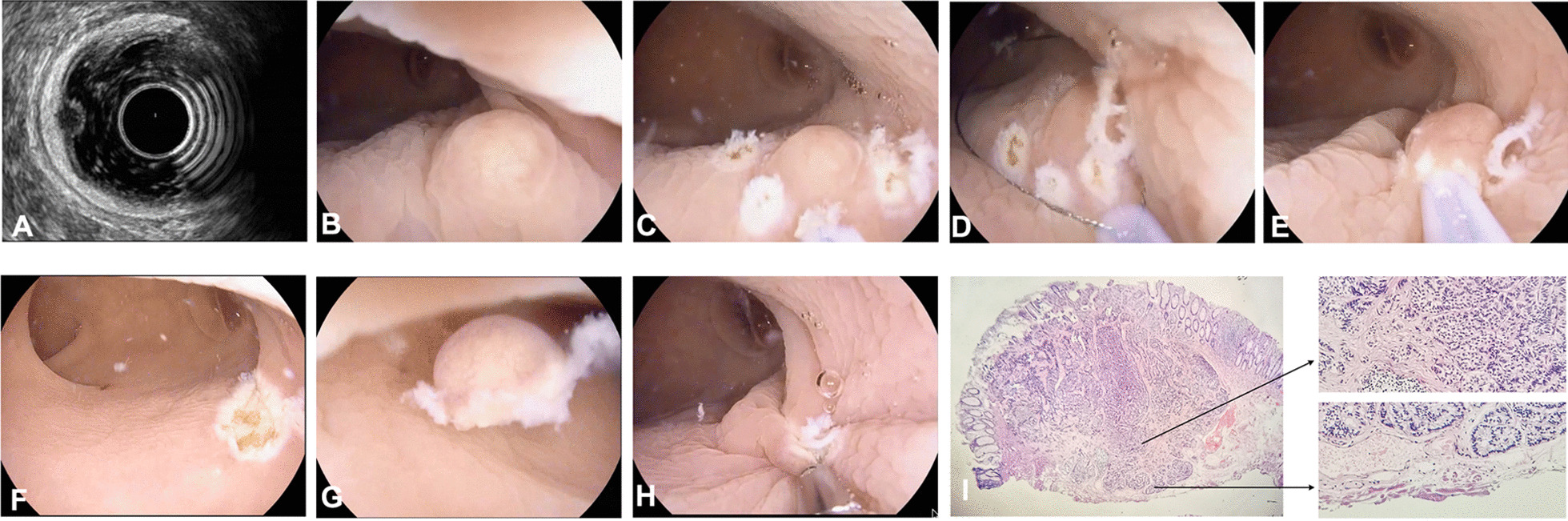
UMER in the treatment of rectal neuroendocrine tumors. **A**: EUS; **B**: the tumor was totally immersed in the water; **C**: the lesion was marked with an argon knife; **D**: the tumor was snared with a 15-mm snare loop and pressed down appropriately; **E**: electrical cutting and coagulation were performed; **F**: the wound; **G**: the resected tumor; **H**: the wound was closed with titanium clips; **I**: histopathology and basal margin

All UEMR were performed by two endoscopists with experience in more than 500 EMR operations. Each patient received standardized bowel preparation prior to endoscopic resection. UEMR was performed using a single-channel electronic colonoscope (PENTAX EC38-i10F) and no anesthesia was required. The procedure included the following steps: the gas in lumen was removed completely and the bowel lumen was fulfilled with distilled water using a water jet pump (AOHUA AFP-1, shanghai, China) until the tumor was completely immersed in the water; argon knife (VIO 200S, ERBE, Germany) was used to mark the lesion 3 mm away from the edge; the tumor was then snared with a 15-mm snare loop (MTN-PFS-E-15, Micro-Tech Nanjing, Nanjing, China) and pressed down appropriately; electrical cutting and coagulation were performed using an Endocut Q current (effect 3, cut duration 2, cut interval 3) and a soft coagulation current (effect 2, 50 W), respectively, which were generated by a high-frequency generator with an automagical control system (VIO 200S, ERBE, Germany); the wound was closed with titanium clips (ROCC-D-26-165, Micro-Tech Nanjing, Nanjing, China); the resected tumors were collected for pathological examination. The UEMR procedure is demonstrated in Fig. 1 and Additional file 1: Video 1.

#### ESD

All ESD were performed by two endoscopists with experience in more than 300 ESD operations. Each patient received standardized bowel preparation prior to endoscopic resection. ESD was performed using a single-channel electronic colonoscope (PENTAX EC38-i10F) after intravenous anesthesia. The procedure included the following steps: argon knife (VIO 200S, ERBE, Germany) was used to mark the tumor 5 mm away from the edge; normal saline mixed adrenaline and methylene blue were injected into the submucosal layer to lift the tumor; the mucosa was incised and the submucosal tissue beneath the tumor was dissected gradually from the muscle layer using a dual knife alone or in combination with a hook knife; electrocoagulation and hemostatic forceps were used when bleeding; the wound was closed with titanium clips (ROCC-D-26-165, Micro-Tech Nanjing, Nanjing, China); the resected tumor were collected for pathological examination.

#### Evaluation of curative effect

The complete operation time was calculated from the colonoscope entering the anus to exiting the anus. The integrity of the specimen was checked after operation to determine whether it was an en bloc resection. The complete resection of the tissue was defined as no residual tumor cells at the horizontal and vertical margins under microscope. Delayed hemorrhage was defined as hemorrhage in the stool within 24 h after operation and required endoscopic hemostasis or surgical hemostasis.

#### Postoperative follow up

The patients were followed up for six months after operation. All patients were rechecked with colonoscopy, biopsy was performed at the suspected recurrence lesions, and pathological diagnosis was performed to confirm each recurrence in situ.

#### Statistical methods

All data were analyzed by SPSS 16.0. The measurement data was expressed by (X ± S), and the independent sample t test was used for comparison; the count data were expressed by [n(%)], and the χ2 test was used for comparison. The difference was statistically significant when *P* ≤ 0.05.

## Results

General information Medical data of 78 patients who underwent colonoscopic rectal neuroendocrine tumor resection at the Endoscopy Center of the Second Affiliated Hospital of Xi'an Jiaotong University from January 2019 to June 2021 were collected. The depth of tumor invasion was confirmed by preoperative endoscopic ultrasonography to ensure all the tumors were confined to mucosa and submucosa. Among them, there were 22 cases in the UEMR group and 56 cases in the ESD group. All cases were pathologically diagnosed with G1 or G2 neuroendocrine tumor based on the 2019 WHO classification [7].

### Comparison of the general information of the two groups

There were no significant differences between the UEMR and ESD groups in gender, age, tumor diameter, distance from the anal margin, and depth of tumor invasion (all *P* > 0.05).(Table 1).

**Table 1:** Comparison of general information between UMER and ESD group

Groups	UEMR (n = 22)	ESD (n = 56)	χ^2^/t	*P*
Gender (n), Male/Female	14/8	30/26	0.651	0.420
Age(years), X ± S	46.4 ± 9.3	47.2 ± 8.7	− 0.352	0.726
Tumor diameter (mm), X ± S	7.2 ± 1.6	6.8 ± 1.7	− 0.883	0.380
Distance from the anal margin (cm), X ± S	7.7 ± 1.6	7.3 ± 1.7	0.963	0.339
Depth of tumor invasion (n), mucosa/submucosa	8/14	14/42	1.007	0.316

### Outcomes of UEMR and ESD in treatment of rectal NETs

The endoscopic complete resection rate of the UEMR and ESD groups were 100%, and there was no statistically significant difference in the histological complete resection rate between the two groups (95.5% vs 96.4%). The vertical margin of all cases in both groups was negative. The horizontal margin was positive in 1 case in the UEMR group and 2 cases in the ESD group respectively. No perforation occurred in the two groups. Delayed hemorrhage occurred in 1 case in the ESD group. Pulsating bleeding was revealed by re-checking the colonoscopy and stopped after being clamped by titanium clips. There was no statistically significant difference in the incidence of delayed hemorrhage between the two groups. The incidence of postoperative abdominal pain and abdominal distention in the UEMR group was lower than that in the ESD group (*P* < 0.05). The operation time, hospitalization time and operation cost of UEMR group were less than those of ESD group (*P* < 0.05). (Table 2).

**Table 2:** Outcomes of UEMR and ESD in treatment of rectal NETs

Groups	UEMR (n = 22)	ESD (n = 56)	χ^2^/t	*P*
Endoscopic complete resection rate, n(%)	22(100)	56(100)	/	/
Histological complete resection rate, n(%)	21(95.5)	54(96.4)	0.041	0.840
Delayed hemorrhage, n(%)	0(0)	1(1.8)	0.398	0.528
Perforation, n(%)	0(0)	0(0)	/	/
Postoperative abdominal pain, n(%)	1(4.5)	14(25.0)	4.255	0.039
Postoperative abdominal distention, n(%)	0(0)	11(19.6)	5.031	0.025
Postoperative anal discomfort, n(%)	1(4.5)	3(5.4)	0.021	0.884
Operation time (min)	5.0 ± 1.4	26.6 ± 8.1	− 12.379	< 0.001
Hospitalization time (d)	4.5 ± 1.1	7.1 ± 1.3	− 8.475	< 0.001
Operation cost (USD)	328.6 ± 20.6	1759.0 ± 72.6	− 134.193	< 0.001

### Comparison of postoperative recurrence rates between the two groups

Each group was followed up for 6 months, and no recurrence cases were found.

## Discussion

According to SEER (Surveillance, Epidemiology, and End Results) database in the United States, the most common location of NETs in the GI tract among patients is the small intestine (38%), followed by the rectum (34%), colon (16%) and stomach (11%) [8]. In Asian group, rectal NETs account for 60% to 89% of all digestive tract NENs in Japan [1], 48% in Korea [9] and 58.93% in China [10]. The total metastasis rate of rectal NETs is 2.3%. The probability of lymph node metastasis in rectal NETs smaller than 1 cm is 1% to 4%, polyps greater than 2 cm and rectal NETs which invade lymph vessels are more likely to metastasize [4]. Therefore, for rectal NETs < 2 cm, if there is no distant or lymph node metastasis and the invasion depth is confined to the mucosa and submucosa according to preoperative endoscopic ultrasound, endoscopic resection or transanal resection can be considered [11]. For patients pathologically diagnosed as G3 NEN after the resection or if the resection is incomplete, surgical treatment should be performed according to the norms of colon adenocarcinoma. Besides, compared to transanal resection, endoscopic resection has the advantage of a less trauma, so it has become an important choice for clinical treatment of rectal NETs.

Endoscopic resection of rectal NETs mainly includes endoscopic mucosal resection (EMR) and endoscopic submucosal dissection (ESD) [12]. EMR is a method of mucosal resection for benign and early malignant lesions of the gastrointestinal tract. The specific operation method is to inject normal saline into the submucosa under the endoscope and form a liquid cushion, so that the lesion to be resected is raised, and the mucosa could be stripped and excised. Theoretically, enough lateral margins of rectal NETs can be removed, but for rectal NETs that invade the deep submucosa, EMR may not achieve complete histopathological resection. Studies have found that the marginal involvement rate of EMR resection is 16%-62% [13]. There are other modified EMR methods including endoscopic submucosal resection with ligation device (ESMR-L), endoscopic mucosal resection with cap (EMR-C) and EMR with circumferential incision (EMR-CI) which might be considered effective and safe methods for treating rectal NETs [14, 15].

ESD is a method of submucosal dissection for precancerous lesions and early cancers of the gastrointestinal tract. Zhong et al. [16]found that ESD is more effective than EMR in complete resection rate (OR = 0.29, 95% CI: 0.14 to 0.58, *P* = 0.000). ESD can control the width and depth of endoscopic submucosal dissection, and control the horizontal and vertical margins more microscopically. It can provide a higher complete resection rate, lower local recurrence rate and more accurate pathological evaluation than EMR. The disadvantage of ESD is that it requires higher operation skills, special equipment, longer operation time, higher operation cost, and takes a higher risk of adverse events such as bleeding and perforation [17]. Some observation studies showed that modified EMR methods including ESMR-L, EMR-C and EMR-CI were superior to ESD in the procedure and operation time [14, 15].

**Fig. 2:**
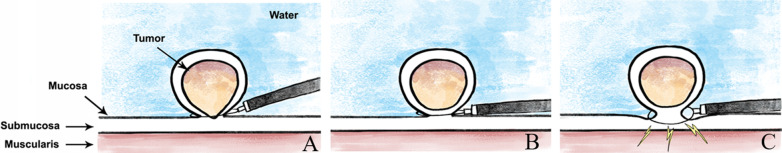
Three ways of snaring and resecting the tumor. **A**: Pull up the snare loop; **B**: Press down the snare loop appropriately; **C**: Press down the snare loop excessively

Underwater EMR technology, which was first reported by Binmoeller et al. [18] in 2012, is turning off the air pump, submerging the lesion by water injection, and snaring to remove the lesion with electricity. Subverting the traditional EMR theory, UMER does not require submucosal injection. After the colon is filled with water and the gas is sucked out, the water pump is used to inject water into the intestinal cavity to completely immerse the lesion. One of its advantages is the elimination of submucosal injections, the intestinal wall maintains its natural thickness of 3 ~ 4 mm (compared to the gas expansion of the intestinal wall to 1 mm). The muscularis propria remains round, and does not fold with mucosa and submucosa. Due to the fat content and the underwater weightless effect, the mucosa appears to float, which is conducive to the complete snare resection of the lesion. While greatly saving operation time and conducive to wound repair, UEMR also greatly reduces the risk of the operation [19]. A multi-center prospective RCT study compared UEMR with conventional EMR for resection of medium-size (10–20 mm) colorectal polyps. The results found that the R0 resection rate of the UEMR group was significantly higher than that of the EMR group, and the en bloc resection rate of UEMR was also significantly higher than the conventional EMR group, while the incidence of adverse events was similar [20, 21]. Since the technology was launched, cases of adenoma resection of the ileum, non-papillae duodenum and appendix orifice have been carried out successively [22–25]. In 2021, some experts reached a consensus on water-assisted colonoscopy and underwater colorectal lesion resection, affirmed the effectiveness and safety of UEMR [6]. There were several clinical evaluations on UEMR in the resection of rectal NETs [26–28], however, those studies did not compare to other type of operations such as ESD, did not follow up properly, and did not examine symptoms and costs. In our study, Both UEMR and ESD can effectively treat ≤ 10 mm rectal NETs with invasion depth confined to the mucosa and submucosa. UEMR is superior to ESD in operation time, hospitalization time, operation cost, postoperative abdominal pain and abdominal distention. Besides, we have made some innovations in our procedure. We routinely mark the edge of the tumor with argon before the operation of UMER, which is conducive to snare the tumor completely. Figure 2 shows three ways of snaring and resecting the tumor, pulling up the snare loop may cause tumor residue at the base level and pressing down the snare loop excessively may cause intestinal wall injury. Therefore, we proposed to press down the snare loop appropriately so that the snare can slide in the plane of the intestinal wall, in order to avoid tumor residue and intestinal wall injury. Retrospectively analyzing the data of 1 UEMR patient with positive horizontal margins, we consider that it may be related to the deeper invasion, the flattened shape of the lesion and pulling up the snare acting while cutting. Meanwhile, both the 2 cases with positive horizontal margins in the ESD group had a deeper invasion. Those 3 cases were all G1 and they are still under close follow-up. At present, there are no direct control studies on UEMR versus EMR-L and EMR-C for treating rectal NETs. However, based on previous literature and our study, it is believed that UEMR does not require additional equipment such as ligatures and transparent caps, and therefore may be easier to operate and take less time to perform.

This study found that UEMR is feasible in the treatment of ≤ 10 mm rectal neuroendocrine tumors located in the mucosal and submucosa layer. The operation method is simple and easy to master. The tumor can be completely removed, with less operation time, hospitalization time and operation cost. However, this study still has certain limitations. It was a single-center retrospective study with a small sample. The histological complete resection rate, complications, and postoperative recurrence still need a larger sample and multi-center study to evaluate.

## Conclusions

Both the UEMR and ESD can effectively treat ≤ 10 mm rectal NETs with invasion depth confined to the mucosa and submucosa. UEMR is superior to ESD in operation time, hospitalization time, operation cost, postoperative abdominal pain and abdominal distention.

## Supplementary Information

**Additional file 1. Video 1.** The procedure of UMER: the gas in lumen was removed completely and the bowel lumen was fulfilled with distilled water using a water jet pump until the tumor was completely immersed in the water; argon knife was used to mark the lesion 3 mm away from the edge; the tumor was then snared with a 15-mm snare loop and pressed down appropriately; electrical cutting and coagulation were performed using an Endocut Q current and a soft coagulation current; the wound was closed with titanium clips and the resected tumors were collected for pathological examination.

## Data Availability

All data generated or analyzed during this study are included in this article. Further enquiries can be directed to the corresponding author.

## Abbreviations

ESD: Endoscopic submucosal dissection
EMR: Endoscopic mucosal resection
UEMR: Underwater endoscopic mucosal resection
NETs: Neuroendocrine tumors
SEER: Surveillance, epidemiology, and end results

## Declarations

### Guidelines and regulations

A ll methods in this study were carried out in accordance with relevant guidelines and regulations.
